# Supplementary material for: Carotenoids from Persimmon (Diospyros kaki Thunb.) Byproducts Exert Photoprotective, Antioxidative and Microbial Anti-Adhesive Effects on HaCaT
Source: Pharmaceutics. 2021 Nov 8;13(11):1898. doi: 10.3390/pharmaceutics13111898 (PMC8618857; doi:10.3390/pharmaceutics13111898)
Supplement: Supplementary file 1 [file pharmaceutics-13-01898-s001.zip › pharmaceutics-1440370-supplementary.pdf]

pharmaceutics

MDPI

Supplementary Materials: Carotenoids from Persimmon (*Diospyros kaki* Thunb.) Byproducts Exert Photoprotective, Antioxidative and Microbial Anti-Adhesive Effects on HaCaT

Sara Gea-Botella, Bryan Moreno-Chamba, Laura de la Casa, Julio Salazar-Bermeo, Nuria Martí, María Concepción Martínez-Madrid, Manuel Valero and Domingo Saura

Table S1. Gradient conditions used for carotenoid analysis.

Time (min)	Flow (mL/min)	Flow Rate	
		A (%)	B (%)
Initial	0.5	40	60
3	0.5	70	30
22	0.5	70	30
26	0.5	90	10
41.5	0.5	90	10
3.5	0.5	40	60

LC column: YMC Carotenoid HPLC Column, C30, 4.6 × 250 mm, 5 µm (Teknokroma Analítica, Sant Cugat del Vallès, Barcelona, Spain); Colum oven temprature at 25 °C; Mobile phase A: methanol/acetone (60/40); Mobile phase B: acetone/water (60/40).

Table S2. MS Parameter settings used for carotenoid analysis: LCMS8050TM (Shimadzu).

ESI	Positive
Capillary (kV)	4.5
IF Temperature (°C)	300
DL Temperature (°C)	200
BH Temperature (°C)	300
Desolvation gas flow (L/min)	5
Nebuliser gas flow (L/min)	3
Desolvation Temperature (°C)	526
Scan Range (*m*/*z*)	100–100

Table S3. Annotation and detection conditions for carotenoid analysis.

Peak	Carotenoid	Ret. Time	λ_{max} (nm)	[M + H]+ (*m*/*z*)	Fragment Ions (*m*/*z*)	CE	Relative Concentration (%)	Franz Cells
1	(All-trans)-lutein	2.224	450/654	552	492,539,268,519	−15	7.306	−
2	zeinoxanthin	2.704	444/455	552	414,485,495,506,493,552	−15	0.740	−
3	(cis)-lutein	3.304	361/455	552	466,184,186,266,235,425	−35	3.063	−
4	luteoxanthin	5.628	400/421	586	528	−15	1.936	−
5	(all-trans)-violaxanthin	6.028	403/471	586	527,568,529,549	−15	1.893	−
6	(cis)-violaxanthin	8.336	319/406	586	364,265,309,295,528	−35	2.005	−
7	(all-trans)-antheraxanthin	10.420	420/470	586	528,474,406,568	−35	8.510	−
8	(cis)-antheraxanthin	13.832	319/438/	586	527,298,540,518	−15	10.319	−
9	β-cryptoxanthin 5,6-epoxide	25.952	420/445	536	176,282,325,386,271	−15	4.157	−
10	5,6-epoxy-α-carotene	27.772	418/445	552	328,280,56,127,110,331,551	−35	1.265	−
11	(all-trans)-zeaxanthin	30.488	417/651	586	527,461	−15	0.852	−
12	(cis)-α-cryptoxanthin	30.724	399/490	552	109,187,304,349,494	−15	0.030	+
13	(cis)-β-cryptoxanthin	31.176	319/421	552	305,459,96,524,177	−15	3.239	+
14	(cis)-α-carotene	31.612	319/490	536	223,268,307,280	−15	0.702	+
15	(cis)-β-carotene	31.928	319/401	536	223,268,307,280	−15	1.221	+
16	(all-trans)-α-carotene	32.364	409/490	536	186,363,162,210	−15	1.784	+
17	(all-trans)-β-carotene	33.216	490/683	536	478,535,400	−15	3.137	+
18	(All-trans)-β-cryptoxanthin	36.764	452/683	553	495,426,460,201,307	−15	44.805	−
19	(all-E)-lycopene	37.808	319/490	536	356,444,192	−15	1.467	−
20	(cis)-lycopene isomer	41.888	319/399	536	318,468,477,223,382	−15	0.529	−
